# Cultural background modulates how we look at other persons’ gaze

**DOI:** 10.1177/0165025412465360

**Published:** 2013-03

**Authors:** Atsushi Senju, Angélina Vernetti, Yukiko Kikuchi, Hironori Akechi, Toshikazu Hasegawa, Mark H. Johnson

**Affiliations:** 1 Birkbeck, University of London, UK; 2 Japan Society for the Promotion of Science, Japan; 3 The University of Tokyo, Japan

**Keywords:** cross-cultural study, eye contact, eye-tracking, face scanning, gaze processing

## Abstract

The current study investigated the role of cultural norms on the development of face-scanning. British and Japanese adults’ eye movements were recorded while they observed avatar faces moving their mouth, and then their eyes toward or away from the participants. British participants fixated more on the mouth, which contrasts with Japanese participants fixating mainly on the eyes. Moreover, eye fixations of British participants were less affected by the gaze shift of the avatar than Japanese participants, who shifted their fixation to the corresponding direction of the avatar’s gaze. Results are consistent with the Western cultural norms that value the maintenance of eye contact, and the Eastern cultural norms that require flexible use of eye contact and gaze aversion.

## Introduction

A brief look at another person’s face would tell you a lot about who they are (e.g., identity, age, gender, ethnicity, health and attractiveness). Faces are also the “window to the soul”, because facial expressions signal their emotional states, and gaze direction would tell you what they see and what they know. Having such significance in human social communication, it is not surprising that faces attract attention from very early in infancy. Even newborns preferentially orient to faces (Farroni et al., 2005; Johnson, Dziurawiec, Ellis, & Morton, 1991), especially those with direct gaze (Farroni, Csibra, Simion, & Johnson, 2002). Eye-tracking studies have demonstrated that infants start to show adult-like face-scanning behaviour, such as preferential fixations on the eyes and mouth (Yarbus, 1967), from as early as 6 weeks after birth (Hunnius & Geuze, 2004). Atypical patterns of face scanning behaviour can be found in neurodevelopmental disorders, such as autism spectrum disorders (ASD), whereby individuals show profound difficulties in social interaction and communication (Senju & Johnson, 2009). Although the mechanisms underlying atypical face-scanning behaviour in ASD is still unclear, it highlights the potential relationship between face-scanning behaviour and the development of social skills.

An important question about the development of face gaze is the role of postnatal environment. Several major theories of social skills development emphasize the role of input from their parents (or caregiver) as well as those from other members of the society, which are essential for the infant brain to learn the social world and become an “expert” (Gauthier & Nelson, 2001; Pascalis et al., 2005). For example, Sugita (2008) reared infant monkeys with no exposure to faces, and found that general preference to faces develops without exposure to faces, but fine discrimination of faces do not develop. The results highlight the role of innate capacity to detect face-like shapes, and the role of postnatal learning in shaping the capacity to recognize individual faces. However, such a control of postnatal environment is impossible in human studies. So, how can we study the effect of postnatal environment on the development of face gaze in humans? One of the most promising ways is a cross-cultural comparison, because different cultural norms would systematically modulate how the people in each culture would learn to process and interact with others in face-to-face communication.

Two independent lines of research contrasted face gaze between Western European/North American culture and Eastern Asian culture, and found clear differences in the face gaze between the two cultures. First, a series of studies (McCarthy, Lee, Itakura, & Muir, 2006, 2008) reported that Canadian participants maintain longer eye contact with an interviewer than Japanese participants when they answer cognitively demanding questions. In these studies, the gaze direction of the participants were analysed from the video recording. These studies clearly show the differential face gaze in realistic face-to-face interaction, which is consistent with the cultural norms that gaze avoidance is perceived as insincere in Western culture, but the same behaviour does not have such a negative value in Eastern culture; it can even signal respect in some contexts (Argyle, Henderson, Bond, Iizuka, & Contarello, 1986). However, the video recording does not have a sufficient spatial resolution to examine which part of the face (eyes, nose or mouth) the participants look at. Second, another series of studies recorded eye movements of Western European (British) and Eastern Asian (mainly Chinese) participants as they processed static images of faces, and found that Western European participants showed triangular fixation on both eyes and mouth, but Eastern Asian participants showed more fixation on the centre of the face (Blais, Jack, Scheepers, Fiset, & Caldara, 2008; Kelly et al., 2011). It was also suggested that reduced fixation on the mouth could partly explain cross-cultural difference in facial expression processing (Jack, Blais, Scheepers, Schyns, & Caldara, 2009). These studies clearly show the subtle differences in face fixations between participants with different cultural backgrounds, but it is not clear whether it is specific to the context they analyse facial information from static images, or a more general pattern of fixations in a more realistic context where they face dynamic sequences of facial actions.

The current study aimed to bridge these gaps in knowledge by investigating how cultural background (British or Japanese) affects the face gaze when they observe dynamic face stimuli. We also examined whether the gaze direction of face stimuli (looking toward or away from the observer) would affect cross-cultural differences in face gaze. We predicted that British participants would maintain longer and sustained eye contact and make a triangular fixation (that is, more fixation on the mouth than Japanese participants), whereas Japanese participants would show shorter and flexible eye fixations and more central fixations. We also predicted that the response to direct and averted gaze would be modulated by the cultural background because of the different cultural norms on the use of eye contact, but no further specific predictions have been made due to the exploratory nature of the manipulation.

## Methods

Nineteen British adults (ten females and nine males, mean age 27.98 years) and 22 Japanese adults (11 females and 11 males, mean age 27.75 years) participated in the study. (Four participants were not included because of excessive eye tracker data loss, under 70% samples.) British adults were recruited in central London, and Japanese adults were recruited in central Tokyo. All the participants have normal or corrected-to-normal acuity.

**Figure 1.**
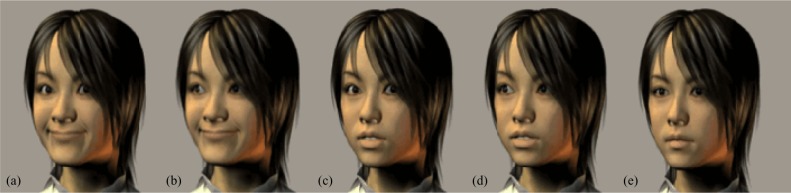
Sample of the gaze direction and mouth movement of the stimuli. *Note.* (a) direct gaze, smile; (b) averted gaze, smile; (c) direct gaze, mouth opening; (d) averted gaze, mouth opening. All the faces were initially presented with (e) forward gaze and closed mouth, which was followed by a mouth movement after 1 second, and a gaze shift after another second (i.e., 2 seconds from the onset of the stimulus). After the gaze shift, the face remained still for another 4 seconds. The orientation of the face was right in half of the stimuli and the left in the other half.

Four computer-generated faces (one Caucasian female, one Caucasian male, one Eastern Asian female, one Eastern Asian male) were selected from the library of Poser 7 (Smith Micro Software, Aliso Viejo, CA), and were used to create 7-second animations with the same software. All the animations started with a face presented upright, facing 30º to the left or to the right and gazing forward, followed by a mouth movement (1 second after the start) and an eye movement (2 seconds after the start). Mouth movements were either smile (Figure 1a, 1b) or mouth opening (Figure 1c, 1d). Eye movements were either direct gaze (Figure 1a, 1c) or averted gaze (Figure 1b, 1d), which involved rotating both eyes laterally by 25º either towards the centre (direct gaze) or away from it (averted gaze). The amount of rotation for direct gaze was selected based on the rating of 10 naïve observers, who rated the perception of “directedness” of the gaze (Todorovic, 2006). The same amount of rotation in the opposite direction was used for averted gaze. In total, 32 animations were generated (4 faces, 2 mouth movements, 2 gaze directions and 2 face orientations). The faces extended 18.4 × 13.0 cm on the screen.

Two Tobii T120 eye-trackers (Tobii, Stockholm, Sweden), equipped with an integrated 17-inch display, were used to present stimuli and record eye-movement in London and in Tokyo. Tobii Studio software was used to control stimulus presentation and to analyse the gaze data.

Recordings were conducted in a quiet and soundproofed room within each research institute. Participants were instructed to watch the movies of the faces. The same experimenter (AS) conducted the recording in both the UK and Japan, to maintain strictly similar experimental conditions such as instructions. A 9-point calibration was conducted using Tobii Studio software before the recording. Recording consisted of two blocks, and each of 32 animations was presented twice (once in each block), in a randomized order. An experimenter also sat in the same testing room, out of sight of the participant, and monitored the recording with Tobii studio software. Viewing distance was approximately 60–65 cm from the display.

**Figure 2.**
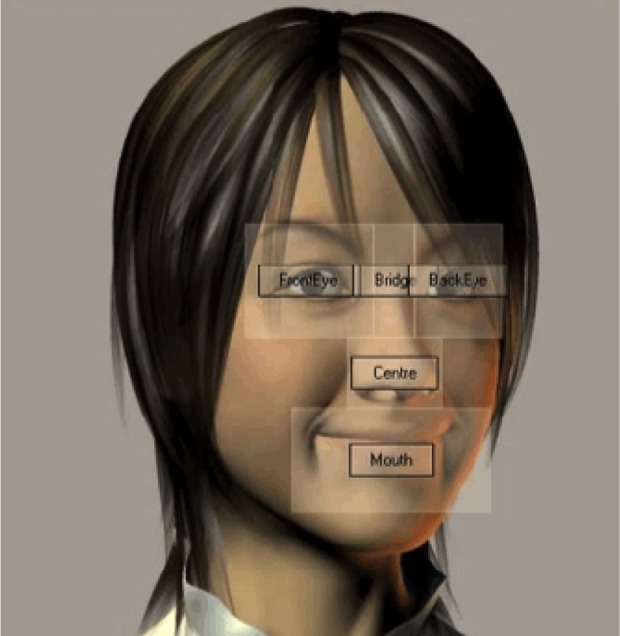
Examples of the area of interest (AOI); front eye; front eye, back eye, bridge, centre and mouth. The size and the location of the AOI were constant across different stimuli.

The gaze data were initially processed with Tobii studio software to calculate the total visit time. Then, we calculated the fixation duration for each stimulus, for the following areas of interest (AOIs); front eye, back eye, bridge, centre and mouth. Note that faces are tilted either to the right or to the left, one of the eyes is always closer to the observer (i.e., Front Eye) than the other eye (i.e., Back Eye), the latter which is off to the side (Figure 2). The AOIs were selected based on the findings of relevant literature in cross-cultural face scanning studies (Blais et al., 2008; Jack et al., 2009; Kelly et al., 2011). These fixation duration data were extracted from the Tobii Studio for statistical analyses.

The gaze data for different head orientations and the blocks were averaged together. The visiting duration for each AOI were then divided with the total visiting duration of the whole face, to calculate the relative visiting duration. The relative visiting duration was analysed with mixed-design ANOVAs to test the effects of cultural background (British or Japanese) and the sex (male or female) of the participants, as well as the ethnicity (Caucasian or Eastern Asian), gender (male or female), gaze direction (direct or averted), mouth movement (smile or mouth opening) and the AOI (front eye, back eye, bridge, centre and mouth) of the stimuli. An initial ANOVA was conducted on the whole 7-second data, which were then followed up by the analyses of seven 1-second bins of the data. For the significant interactions, post-hoc analyses were conducted on each contrast with Wilcoxon sign rank tests with the Bonferroni correction for multiple testing, to provide robust statistics.

## Results

As predicted, the interaction between the cultural background, gaze direction and the AOI was significant, *F*(4, 148) = 4.684, *p* < .01, *η_p_^2^* = 0.11, demonstrating that British and Japanese participants fixated differently to the face, depending on whether the face was with direct or averted gaze. The effect was modulated by the sex, *F*(4, 148) = 4.142, *p* < .01, *η_p_^2^* = 0.10 (see also the supplementary material), but not with other factors such as the mouth movement, the gender or the ethnicity of the face stimuli. These interactions remained significant when we excluded British participants of non-Caucasian ethnic origin (three females), and when we excluded British participants who had stayed two years in East Asian countries (two males, one in China and the other in Thailand) and East Asian participants who had stayed 6 months in the USA (two males).

**Figure 3.**
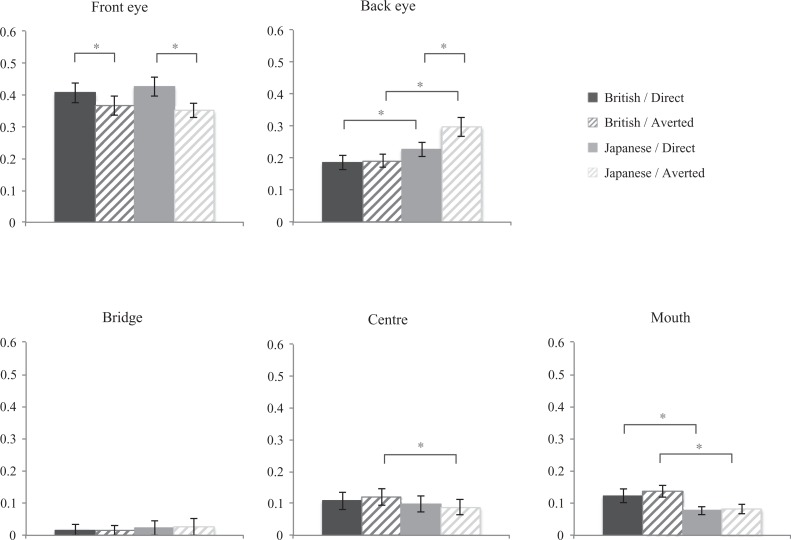
Relative visiting duration on each AOI during the entire period of stimulus presentation, for each cultural background of the participants and the gaze direction of stimuli. *Note.* * *p* < .05 (corrected); error bar: standard error.

Follow-up analyses revealed that Japanese participants fixated longer on the back eye than did British participants, in both direct gaze and averted gaze conditions. British participants, by contrast, fixated longer on the mouth than Japanese participants, in both direct gaze and averted gaze conditions. British participants also showed longer fixation on the centre, which was only significant in the averted gaze condition (Figure 3).

The two groups showed similarities and differences in the response to different gaze directions. Both groups showed longer fixations on the front eye in the direct gaze condition than in averted gaze condition. However, only Japanese participants showed differential fixations on the back eye, with longer fixation in averted gaze condition (Figure 3). No other contrasts reached significance, including any contrast in the bridge area.

Further analyses were conducted on the seven 1-second bins of the data, to explore the time-course of the differential fixations on the four AOIs showing the gaze and cultural background interactions; front eye, back eye, mouth and centre.

### Front eye

**Figure 4.**
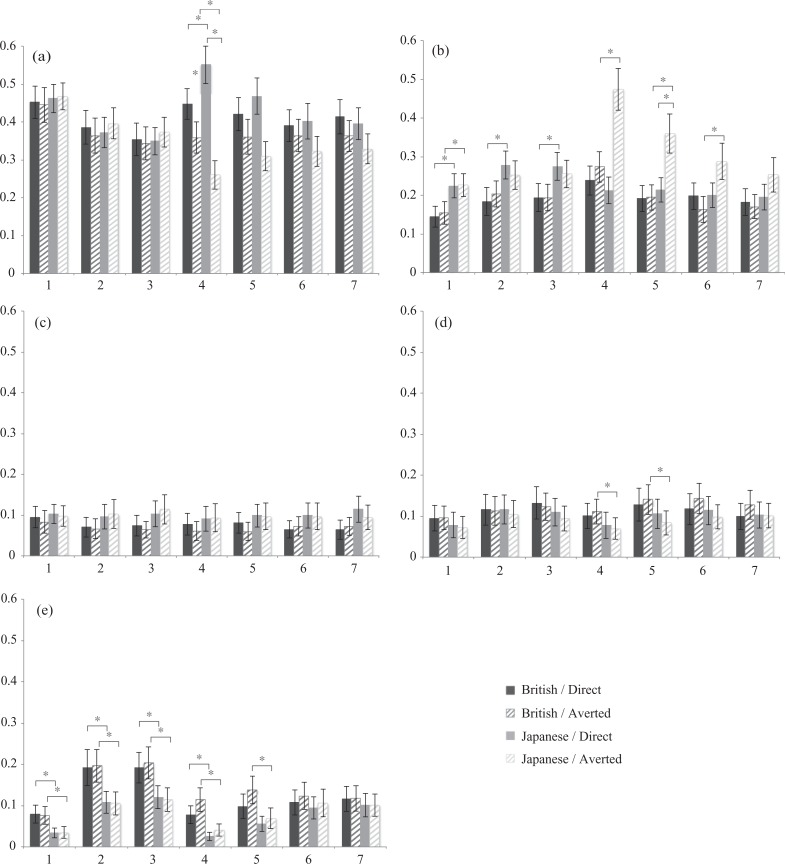
Relative visiting duration on each AOI for each 1-second bin of stimulus presentation, for each cultural background of the participants and the gaze direction of stimuli. *Note.* (a) front eye, (b) back eye, (c) bridge, (d) centre and (e) mouth. The mouth movement occurred during the second bin, and the eye movement occurred during the third bin. * *p* < .05 (corrected); error bar: standard error.

No effects reached significance for the first, second and third bins. In the fourth bin, the point right after the gaze shift of the stimuli, both groups showed longer fixation in direct gaze condition than in averted gaze condition. Interestingly, this effect was exaggerated in Japanese participants, who showed even longer fixation than British participants in response to direct gaze and even shorter fixation than British participants in the averted gaze condition (Figure 4a). The same trend remained in the fifth bin, in which only Japanese participants looked longer in the direct gaze condition than in the averted gaze condition. No effect reached significance from the sixth bin.

### Back eye

In the first, second and third bins, the only significant effects were the differences between groups, with Japanese participants fixating longer than British participants. From the fourth bin onward, however, Japanese participants fixated longer than British participants only in the averted gaze condition, but not in the direct gaze condition (Figure 4b).

### Mouth

The group difference in mouth fixation remained from the first to the fourth bin (Figure 4e), which then became only significant in averted gaze condition in the fifth bin and non-significant from the sixth bin.

### Centre

The group differences were significant in the fourth and fifth bins, only in averted gaze condition (Figure 4d).

## Discussion

The current study is the first to investigate how the cultural background of the observer affects the face gaze when they observe dynamic faces looking toward or away from the observer. The results clearly demonstrate the cultural difference between British and Japanese participants in the way they look at different parts of the faces, and how they respond to different gaze direction. First, British participants fixated more on the mouth than did Japanese participants, replicating previous studies using static images of faces (Blais et al., 2008; Jack et al., 2009; Kelly et al., 2011). Second, both groups of participants fixated equally long on the eye closer to the observer, but Japanese fixated longer on the other eye than British participants. It is consistent with the finding that Eastern Asian participants fixate longer on the eyes (Jack et al., 2009) but it is a novel finding because to our knowledge, this study is the first to use head-turned faces. On the other hand, we did not replicate the longer fixation on the central parts of the faces in Eastern Asian participants, which is reported in previous studies (Blais et al., 2008; Kelly et al., 2011). By contrast, we even found a longer fixation in British participants on the centre in averted gaze condition (Figure 4d), which happened later than the increased mouth fixation (Figure 4e) and is possibly explained by the residual effects of fixations on the mouth. It might suggest that the increased central fixation on the face in the Eastern Asian population is specific to the task which requires perceptual analyses of the faces, such as the recognition and categorization tasks used in the previous studies (Blais et al., 2008; Kelly et al., 2011), and does not happen in passive viewing. Another possibility is that Eastern Asians look more to the eyes when the face is expressive, but fixate centrally when they observe faces with neutral expression. Moreover, Japanese participants did not show shorter eye fixation than British participants, which is consistent with some studies (e.g., Jack et al., 2009) but not with others (McCarthy et al., 2006, 2008). Further studies will be required to test whether the shorter face gaze could be observed in Eastern Asian participants in more naturalistic settings.

Interestingly, these two groups responded differently to the gaze shift of the stimuli. Second-by-second analyses revealed that both groups of participants fixated longer on the front eye immediately (that is, around 1 second) after they saw the gaze shift toward them, but such a change was more exaggerated and lasted longer with Japanese participants than British participants. Moreover, the initial cultural difference in the fixation on the back eye was overridden by the effect of gaze shift, in which Japanese participants fixated longer on the back eye only when they saw an averted gaze. By contrast, the fixation on the back eye was not affected by the gaze direction in British participants. The results showed that Japanese participants shifted their own fixation to the corresponding direction of the observed gaze shift (i.e., to the front, medial eye in response to direct gaze and to the back, lateral eye in response to averted gaze), as if they “followed” the direction of the face gaze. British participants did not show such a change of fixation following observed gaze shift. It might suggest that the eye fixation of British participants reflects the cultural expectation to maintain eye contact, but the eye fixation of Japanese participants reflects the cultural norm to conform to others’ behaviour. Note that it is not a general difference in the sensitivity to facial motion, because mouth movements did not exaggerate or diminish the cultural differences in mouth fixation. The effect was more prominent in males than in females (see the Supplementary material), suggesting that the effect of cultural norm on face and gaze processing manifests more strongly in males. Further studies will be required to see how the differences in gender-related cultural norm interact with face-scanning behaviour.

The current study clearly demonstrates the cultural differences in face gaze in adults, but it cannot tell us how it develops. For example, Kelly et al. (2011) demonstrated that 70% of British-born Chinese adults show a face fixation pattern similar to Eastern culture, whereas 30% of them show a Western pattern of face fixation. This study suggests that cultural diversity in face fixation is more strongly affected by early familial environment, but could also be affected by societal environment (e.g., peers) in some individuals. Further studies will be necessary to study the time-course of the emergence of cultural diversity in face gaze early in the development. Moreover, as in previous adult studies (Blais et al., 2008; Jack et al., 2009; Kelly et al., 2011), we did not replicate the other race effect on face scanning (that is, the significant interaction between the ethnicity of the participants and the ethnicity of the stimuli), which contrasts with previous infant research (Liu et al., 2011; Wheeler et al., 2011). Future developmental studies will be essential to assess the role of face familiarity on face scanning throughout the course of development. We also need to examine how the gender difference found in the current study (that is, larger cross-cultural differences in male than in female participants) develops, by testing younger populations.

To summarize, the current study revealed that the cultural background of the participant affects how they look at another person’s eyes and mouth, and how they modulate eye fixation in response to the gaze direction of others. These differences are consistent with the culturally-relevant strategy of perceptual analyses, as well as the cultural norms on the use of eye contact in face-to-face communication. These results highlight the new frontier of the research about how cultural norms can affect behavioural, cognitive and neural development, which would provide a great opportunity to study the effect of postnatal environment on human behavioural and cognitive development.

## References

[bibr1-0165025412465360] Argyle M. Henderson M. Bond M. Iizuka Y. Contarello A. (1986). Cross-cultural variations in relationship rules. International Journal of Psychology, 21, 287–315

[bibr2-0165025412465360] Blais C. Jack R. E. Scheepers C. Fiset D. Caldara R. (2008). Culture shapes how we look at faces. PLoS ONE, 3, e3022 18714387 10.1371/journal.pone.0003022 PMC2515341

[bibr3-0165025412465360] Farroni T. Csibra G. Simion F. Johnson M. H. (2002). Eye contact detection in humans from birth. Proceedings of the National Academy of Science of the United States of America, 99, 9602–9605 10.1073/pnas.152159999 PMC123187 12082186

[bibr4-0165025412465360] Farroni T. Johnson M. H. Menon E. Zulian L. Faraguna D. Csibra G. (2005). Newborns’ preference for face-relevant stimuli: Effects of contrast polarity. Proceedings of National Academy of Science of the United States of America, 102, 17245–17250 10.1073/pnas.0502205102 PMC1287965 16284255

[bibr5-0165025412465360] Gauthier I. Nelson C. A. (2001). The development of face expertise. Current Opinion in Neurobiology, 11, 219–224 11301243 10.1016/s0959-4388(00)00200-2

[bibr6-0165025412465360] Hunnius S. Geuze R. H. (2004). Developmental changes in visual scanning of dynamic faces and abstract stimuli in infants: A longitudinal study. Infancy, 6, 231–255 10.1207/s15327078in0602_5 33430528

[bibr7-0165025412465360] Jack R. E. Blais C. Scheepers C. Schyns P. G. Caldara R. (2009). Cultural confusions show that facial expressions are not universal. Current Biology, 19, 1543–1548 19682907 10.1016/j.cub.2009.07.051

[bibr8-0165025412465360] Johnson M. H. Dziurawiec S. Ellis H. Morton J. (1991). Newborns’ preferential tracking of face-like stimuli and its subsequent decline. Cognition, 40, 1–19 1786670 10.1016/0010-0277(91)90045-6

[bibr9-0165025412465360] Kelly D. J. Jack R.E. Miellet S. De Luca E. Foreman K. Caldara R. (2011). Social experience does not abolish cultural diversity in eye movements. Frontiers in Psychology, 2: 95 doi: 10.3389/fpsyg.2011.00095 21886626 10.3389/fpsyg.2011.00095 PMC3154403

[bibr10-0165025412465360] Liu S. Quinn P. C. Wheeler A. Xiao N. Ge L. Lee K. (2011). Similarity and difference in the processing of same- and other-race faces as revealed by eye tracking in 4- to 9-month-olds. Journal of Experimental Child Psychology, 108, 180–189 20708745 10.1016/j.jecp.2010.06.008 PMC3740558

[bibr11-0165025412465360] McCarthy A. Lee K. Itakura S. Muir D. W. (2006). Cultural display rules drive eye gaze during thinking. Journal of Cross-Cultural Psychology, 37, 717–722 19122788 10.1177/0022022106292079 PMC2613330

[bibr12-0165025412465360] McCarthy A. Lee K. Itakura S. Muir D. W. (2008). Gaze display when thinking depends on culture and context. Journal of Cross-Cultural Psychology, 39, 716–729

[bibr13-0165025412465360] Pascalis O. Scott L. S. Kelly D. J. Shannon R. W. Nicholson E. Coleman M. Nelson C. A. (2005). Plasticity of face processing in infancy. Proceedings of National Academy of Science of the United States of America, 102, 5297–5300 10.1073/pnas.0406627102 PMC555965 15790676

[bibr14-0165025412465360] Senju A. Johnson M. H. (2009). Atypical eye contact in autism: Models, mechanisms and development. Neuroscience & Biobehavioral Reviews, 33, 1204–1214 19538990 10.1016/j.neubiorev.2009.06.001

[bibr15-0165025412465360] Sugita Y. (2008). Face perception in monkeys reared with no exposure to faces. Proceedings of the National Academy of Sciences of the United States of America, 105, 394–398 18172214 10.1073/pnas.0706079105 PMC2224224

[bibr16-0165025412465360] Todorovic D. (2006). Geometrical basis of perception of gaze direction. Vision Research, 46, 3549–3562 16904157 10.1016/j.visres.2006.04.011

[bibr17-0165025412465360] Wheeler A. Anzures G. Quinn P. C. Pascalis O. Omrin D. S. Lee K. (2011). Caucasian infants scan own- and other-race faces differently. PLoS ONE, 6(4), e18621 21533235 10.1371/journal.pone.0018621 PMC3076379

[bibr18-0165025412465360] Yarbus A. (1967). Eye movement and vision. New York, NY: Plenum

